# The mere-measurement effect of patient-reported outcomes: a systematic review and meta-analysis

**DOI:** 10.1007/s11136-025-03909-y

**Published:** 2025-02-05

**Authors:** Preston A. Long, Anouk S. Huberts, Anouk Neureiter di Torrero, Lisa R. Otto, Alizé A. Rogge, Valentin Ritschl, Tanja A. Stamm

**Affiliations:** 1 Institute for Outcomes Research, Center for Medical Data Science, Medical University of Vienna, Spitalgasse 23, 1090 Wien, Austria; 2 Department of Quality and Patient Care, Erasmus Medical Center Rotterdam, Rotterdam, The Netherlands; 3 Center for Patient-Centered Outcomes Research, Charité – Universitätsmedizin Berlin, Corporate Member of Freie Universität Berlin and Humboldt-Universität zu Berlin, Berlin, Germany; 4 Department of Psychosomatic Medicine, Charité – Universitätsmedizin Berlin, Corporate Member of Freie Universität Berlin and Humboldt-Universität zu Berlin, Berlin, Germany; 5 Ludwig Boltzmann Institute for Arthritis and Rehabilitation, Vienna, Austria

**Keywords:** Mere-measurement effect, Question-behaviour effect, Patient-reported outcomes, Systematic literature review

## Abstract

**Purpose:**

The mere-measurement effect is the phenomenon in which subjects exposed to measurements have their perceptions and/or behaviors on the inquired topic affected simply through the act of responding. Patient-reported outcomes (PROs) are increasingly used to assess patient perspective and quality of life in clinical trials and different health care settings. This systematic literature review aims to assess what is currently known about the mere-measurement effect of PROs.

**Methods:**

A systematic literature review and meta-analysis was conducted. We included studies that provided evidence on perceptual or behavioral changes in patients as a result of exposure to questionnaire items assessing PROs. All adult participants were included regardless of demographics. Any study design was considered eligible for inclusion. The databases MEDLINE [PubMed], CINAHL [Ebsco], Web of Science and ScienceDirect were searched.

**Results:**

The search resulted in 636 articles which led to a final extraction of nine. Overall, seven of the nine articles reported a significant main effect, i.e. presence of the mere-measurement effect. For the meta-analysis, thirteen different interventions were included. There was a one-directional, positive and significant overall risk ratio of 1.17 [CI95% 1.04;1.30].

**Conclusion:**

This systematic review found significant potential for the mere-measurement effect to shape respondents’ behaviors or perceptions for the better, opening the door to the possibility of engineering PROs to serve as a subtle intervention. Future considerations and directions for research are discussed.

**Supplementary Information:**

The online version contains supplementary material available at 10.1007/s11136-025-03909-y.

## Plain english summary

Patient-reported outcome measures are increasingly collected in clinical settings for multiple purposes, including assessing the efficacy of new interventions, comparing population groups, assessing the quality of care delivered to patients, and understanding the trajectories of changes in patient outcomes over time. While this is generally a positive trend, the impact of exposing patients repeatedly to health-related questionnaires has been largely neglected. For example, there is evidence to suggest that asking a patient about their intent to receive a treatment can actually increase the odds they do so. A systematic literature review and follow-up statistical assessment of the overall impact was conducted to assess what is currently known about the effect of presenting questionnaires to people. For example, how does emotional wording of a question or statement impact a person’s perception or behaviour. The results of this study show that merely asking questions can have an overall meaningful effect on a patient’s behavior or perception on the topic they were asked about. It is also clear that the potential positive and negative effects of taking measurements, and methods to reduce these effects or to harness the positive, have not been widely considered in the medical sciences.

## Introduction

Patient-reported outcomes (PROs) are any type of response on a patient’s health and wellbeing which is sourced directly from the patient without any outside interpretation [1, 2].These types of outcomes span numerous domains from mental health, to quality of life, to pain perceptions, and are most commonly collected in clinical settings. The use of PROs is also becoming increasingly widespread and may soon become a required component of clinical drug trials in the EU as supported by the European Medicine Agency (EMA) [3]. PROs offer the possibility to increasingly personalize treatments, assess labelling claims and aid quality assurance [4–6].

In order to ensure a valid measurement, psychometric rigor is applied in the development of PRO measures (PROMs). However, there is a subtle and potentially both insidious and benevolent element of PROs which to date has received little attention: the mere-measurement effect. This effect, also known as the question-behavior effect or assessment reactivity effect, denotes the act of measurement’s ability to influence and transform responders thoughts, attitudes, and actions [7]. Acknowledged in cognitive psychology, behavioral economics, and social psychology, this phenomenon highlights the innate impact that the exposure to questions and statements have on shaping decisions and behaviors.

At its core, the mere-measurement effect postulates that the act of measuring a particular aspect of human behavior or attitudes can exert a distinct influence, causing subsequent changes in the very behaviors or attitudes being measured [8]. The very act of considering and responding to measure items can activate cognitive mechanisms that nudge individuals toward aligning their actions with their stated intentions, often without the need for explicit external interventions [9]. For instance, if patients report that they can do household chores without issue, they may be more likely to actually live up to their self-report. Research has shown that the effect remains present even through implicit measurements which were designed to circumnavigate conscious awareness [10].

Furthermore, the mere-measurement effect touches upon the realms of self-perception and identity construction. When individuals are prompted to evaluate their own attitudes, preferences, or beliefs, the process can lead to a reinforcement of these aspects within their self-concept [11]. Consequently, the act of measurement contributes not only to revealing pre-existing attitudes but also to shaping an individual's sense of identity, like a reflection we become primed to fulfill. Research has further expanded on this explanation by demonstrating that responding with socially undesirable intentions can increase the likelihood of behavioural follow through by offering the responder self-permission to “sin”, if the questions do not highlight the negatives of the behaviors within their text [12].

There are two leading complimentary theories which explain this effect, the behavioral stability hypothesis and the context stability hypothesis. The behavioral stability hypothesis as it relates to the mere-measurement effect posits that routine behaviors conducted in routine contexts are less susceptible to the mere-measurement effect [13]. Whereas, measurements about future intentions are more likely to influence uncommon behaviors. The context stability hypothesis claims that measurements involving uncommon scenarios increase the mere-measurement effect [14]. In summary, the degree and direction of effect seems dependent on the alignment (or lack thereof) of the past behaviors, intentions, and contexts. For example, asking students about their drinking habits before they went into a bar (consistent context), the drinking behavior went up, while asking students the same questions but prior to entering a lesser paired context like a home, the behavior went down [15].

These theories attempt to account for a primary components of the mere-measurement effect, although there are other possible factors which feed into the directionality and degree of impact of the mere-measurement effect. Likely factors include: emotional valence (positive or negative), number of exposures, and consistency of exposures. For instance, over exposure to a measurement can show a curvilinear trend reversing direction of influence overtime [16]. The impact of the mere-measurement effect can also be positive or negative. Individuals who are asked to express their intentions to engage in healthier lifestyles, such as exercising more, may subsequently find themselves more motivated to adhere to these intentions, however asking about unhealthy behaviors such as drug-use can elicit the same increase [17].

There is one measurement domain in which arguably the mere-measurement effect deserves particular attention: patient-reported outcomes (PROs). These outcomes span numerous domains from mental health, to quality of life, to pain perceptions, and are most commonly collected in the clinical setting. As the use of PROs becomes standard, the unknown effect of their collection must be considered. Although to date, there is a paucity of studies conducted with this context in mind. Hence, the purpose of this systematic literature review is to identify any studies which focused on the mere-measurement effect of PROs and capture what is currently know.

In the context of PRO assessment, the mere-measurement effect and possible factors which feed into the directionality have rarely been investigated. As PRO use rises, their impact on the patients must be further examined. In order to begin filling in this knowledge gap, this systematic literature review sought to answer four questions. (1) Does the positive or negative wording of items affect the patient’s perspective on the latent topic variable? (2) Is there a degree of subliminal influence or measurement effects on their behavior resulting from exposure to PROs? (3) Is such an effect amplified with repeated exposure? (4) Do studies currently examine the effect with PROs as described in this study?

## Methods

The present systematic review was registered in medRxiv prior to the literature search and the protocol was published on March 30, 2022 [18] (medRxiv 2022.03.29.22273094; https://doi.org/10.1101/2022.03.29.22273094). Two modifications occurred after registration: an extra researcher was included in the research team, and grey literature research was conducted. We followed the Preferred Reporting Items for Systematic Reviews and Meta-Analyses (PRISMA) guidelines [19] where applicable and used the PRISMA checklist to ensure rigor in conducting and the reporting of this systematic review.

### Data sources and search strategy

We searched the following databases until September 13th, 2022: MEDLINE [PubMed], CINAHL [Ebsco], Web of Science and ScienceDirect. The following search terms were utilized: "Adult" AND "Intention" [MeSH] AND "Surveys and Questionnaires" [MeSH] self-report "Emotions" [MeSH] AND "Health Behaviour" [MeSH]] AND "Psychometrics" [MeSH] “Humans” [MeSH] AND "self-reports" [MeSH] AND "subjective symptomatology" [MeSH]. A follow-up search was conducted to fill the potential gap between October 2022 and August 2024. Five new articles were identified but resulted in no new inclusions. The search strategy was developed and refined through an iterative process consisting of three blocks: (1) various terms for mere-measurement, (2) various terms for patients and (3) and various terms for health behaviors (Table 1). We identified additional studies via a grey literature search by screening the identified systematic reviews in our search which entailed mere-measurement without focus on patients.

**Table 1:** Search terms for the mere-measurement effect

PsyInfo-Terms (exploding all trees) for mere-measurement in PROs	
“Mere-exposure, Question-behaviour, Nudge, Attitudes, Exposure” Mere-measurement, Subliminal stimulation, emotional responses, stimulus frequency, attitude change, behaviour change, health behaviour, decision making, choice behaviour, attitude formation, affective valence, emotional content	

### Study selection

We included studies that provided evidence on perceptual or behavioral changes in patients as a result of exposure to PROs, the mere-measurement effect. An exposure to the mere-measurement effect was defined as a purposeful study measurement in which the effect of responding was assessed. The following, internally developed definition of patient was used: subjects in the study were in a healthcare setting during participation, and/or, responding to items about their illness status (i.e. treatment, recovery, etc.), or healthcare decisions (including hypotheticals and vignettes). This definition was kept purposefully broad as a quick search revealed few relevant studies for a narrower definition of patients. Additionally, PROs as an inclusion criterion were defined as: outcomes derived directly from participant input which is collected in the context of healthcare, and/or the implemented questionnaire’s focus is directly related to a responder’s clinical diagnoses. All settings were considered, such as home care, community services, primary health care, hospital settings. All adult participants were included regardless of demographics. Studies were excluded if they included non-patient population, did not assess the mere-measurement effect following an initial measurement (e.g. used cross-sectional data), were not written in English, and when they described the development of new psychometric methods, assessments, or screening tools. All articles were imported and screened using Rayyan-AI powered tool for systematic reviews [20]. All articles included in any systematic reviews identified in this search were also reviewed for inclusion.

Four interdisciplinary healthcare researchers (AH, PL, AR, LO) independently screened all titles and abstracts obtained from the search. Subsequently, the eligibility of the full text articles was independently explored by the same four reviewers. Disagreements, revealed after un-blinding, were resolved via consensus meetings.

### Data extraction and synthesis

The following data were extracted from eligible studies by two reviewers independently (PL and AH): authors, country, funding, study design, disease, setting, inclusion and exclusion criteria, collection points and timing, sample size, % female of control group and intervention group, mean age control group and intervention group, primary and secondary interventions, perceptual outcomes, behavioral outcomes, emotional valence (positive, negative or neutral framing of an items wording), primary and secondary instrument/scale, results, additional results, conclusion and limitations. All results that were compatible with each outcome domain in each study were sought. Any disagreements were discussed until consensus was reached. Information that was missing or unclear and which couldn't be resolved was marked as missing.

### Quality assessment (QA)

Two reviewers (PL and ANT) independently assessed the methodological quality of included studies. We used the Effective Public Healthcare Panacea Project (EPHPP)’s Quality Assessment Tool for Quantitative Studies [21]. The rounded average score of six components, selection bias, study design, confounders, blinding, data collection methods and withdrawals and drop-outs, determined the global rating.

### Meta-analysis

Following completion of the data extraction, a meta-analysis was performed on the included articles to calculate an approximate effect size of mere-measurement. All interventions, sourced from the qualifying articles, which included a control condition, were included. An overall risk ratio was then generated using R including the following packages: *metafor* package [22] (v2.4-0; Viechtbauer, 2010) *escalc* function, *dmetar* package [23] (v0.1.0; Harrer, 2019), and *meta* package [24] (v4.17-0; Balduzzi, 2019). The corresponding visuals were created using the forest function from the meta package [25].

For the purpose of the meta-analysis, a random effect model was assumed. To ensure the comparability and interpretability of the results, all effect sizes underwent a log transformation before generating the confidence intervals in order to correct for the non-parametric nature of risk ratios [25]. A study in which the risk ratio's confidence interval's min and max are both above 1.0 should be interpreted as a significant increase in odds, i.e. that the mere-measurement effect impacted respondents [26]. Due to the positive homogeneity of the interventions, the directionality of every significant risk ratio implies a positive manipulation occurrence in participants.

A test for heterogeneity was also performed using an inverse variance method and Paule-Mandel estimator for tau^2^ (see details below). Inclusion criteria for meta-analysis included: have a control, count an intervention only if it is measured with a direct behavioural or perceptual outcome, report relative frequencies of intervention and control subjects with significant effect, report exposures if shown in the reviewed studies, subjects can be reasonably considered patients as defined in the protocol, and explicitly mention question-behaviour effect, mere measurement effect or assessment reactivity. If a study didn’t have a control group, they were excluded from the meta-analysis. Missing data from systematic review variables that were extracted were left blank. The parameters of Meta-analysis were as follows: standardized effect sizes were calculated for each reported outcome; heterogeneity was assessed using Cochrane’s test, where a Q statistic (Chi-square) determined whether the effect sizes significantly differed. P-value of the Q test of 0.05 or lower indicated the presence of heterogeneity; if heterogeneity was detected, Higgins I2 was used to measure its extent. An I2 value of 50% or higher suggested substantial heterogeneity, leading to the application of a random-effects model; outlier analysis was conducted to identify and exclude studies causing significant heterogeneity. The meta-analysis was then recalculated.

To identify potential publication biases, funnel plots were employed. A wider scatter of smaller studies around the mean effect size compared to larger studies indicated bias. Asymmetric distribution in the funnel plot suggested publication bias.

## Results

**Fig. 1:**
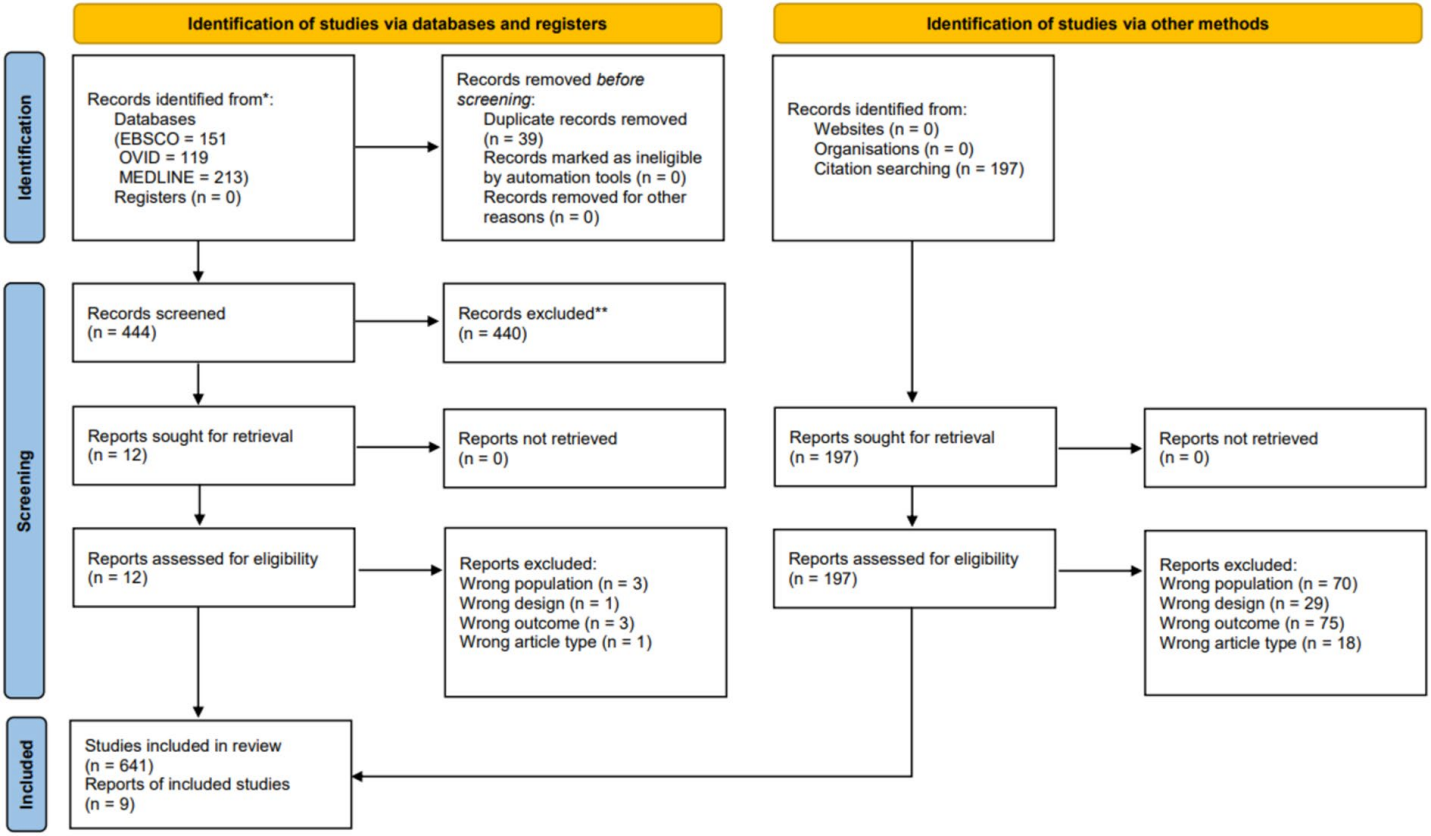
Process of the systematic literature review (PRISMA flow diagram)

The search resulted in 636 abstracts which led to a final extraction of 9 articles. A PRISMA flow diagram is presented in Fig. 1. The quality assessments (QA) produced two disagreements which were resolved, concluding in five moderate and four strong studies. No weak studies were identified suggesting a low risk of bias. Six of these nine (66%) articles were conducted in the United Kingdom [27–32], the other three were conducted in either The Netherlands [33], Poland [34] or the United States [35]. The majority of the studies evaluated items that were solely neutrally worded (56%) with the remainder roughly equally split between positive and negative wording leaving inadequate group sizes for assessment. Importantly, the positive and negative valences were accidental artefacts of the items asked and were not designed purposefully to test the sub-effect of emotional valence. All but one of the mere-measurement outcomes were behavioural (89%). While the medical areas were diverse, seven interventions focused on increasing health check-up or treatment rates in their respective field. Though the studies included PROs, only one article explicitly used the term “patient-reported outcome measure” or PROM. Every study did, however, use self-reported questionnaires. Only one study used an objective intervention (accelerometer) in combination with a self-report.

Overall, seven of the nine articles reported finding a significant main effect, i.e. presence of the mere-measurement effect [28–33, 35]. Four studies demonstrated that completing a questionnaire increased the attendance rates of vaccinations, health checks or cervical screening [28, 30, 32, 35]. The study conducted by Cartwright et al. (2012) showed that questionnaire context influenced responses on measures of illness impact [29]. One study showed that assessing levels and determinants of physical activity affected participants’ physical activity behaviour [33]. Ayres et al. (2013) showed an increase in obtaining a health plan when a motivational intervention was combined with a questionnaire [31] (Table 2).

**Table 2:** Results of factor extraction from included articles

Article	Country	Design	Primary intervention	Primary outcome	Emotional valence	Quality assessment	MM effect
			* Self-reported				
Conner, 2017	United Kingdom	RCT	*Intent assessment	Behavioral: Vaccination rates	Negative and Positive	Strong	Found
Shafran, 2018	United Kingdom	RCT	*Mental health symptom monitoring	Behavioral: Treatment receipt	Neutral	Moderate	Not Found
Sandberg, 2010	United Kingdom	Prospective randomized	*TPB questionnaire	Behavioral: Attendance rates	Negative	Strong	Found
Cartwright, 2011	United Kingdom	Prospective randomized	*Perceived impact measure	Perceptual: Change in perceived impact	Neutral	Strong	Found
Cherpitel, 2010	Poland	RCT	*Drinking habits	Behavioral: Treatment receipt	Negative and Neutral	Moderate	Not Found
van Sluijs, 2005	Netherlands	RCT	*Physical activity and wearing an accelerometer	Behavioral: Self-reported and objectively measured physical activity	Neutral	Moderate	Found
Conner, 2011	United Kingdom	RCT	*TPB questionnaire	Behavioral: Attending health check	Positive	Strong	Found
Cox, 2011	United States	RCT	*Short-term barriers to vaccinations	Behavioral: Vaccination rates	Neutral	Moderate	Found
Ayres, 2011	United Kingdom	RCT	*Health action plan	Behavioral: Creating a health plan	Neutral	Moderate	Found

*MM* Mere-measurement, *RCT* randomized clinical trial, *TPB* theory of planned behavior

For all outcomes for each study summary statistics for each group (where appropriate) and an effect estimate and its precision are presented in Supplement Table 1.

### Meta-analysis

**Fig. 2:**
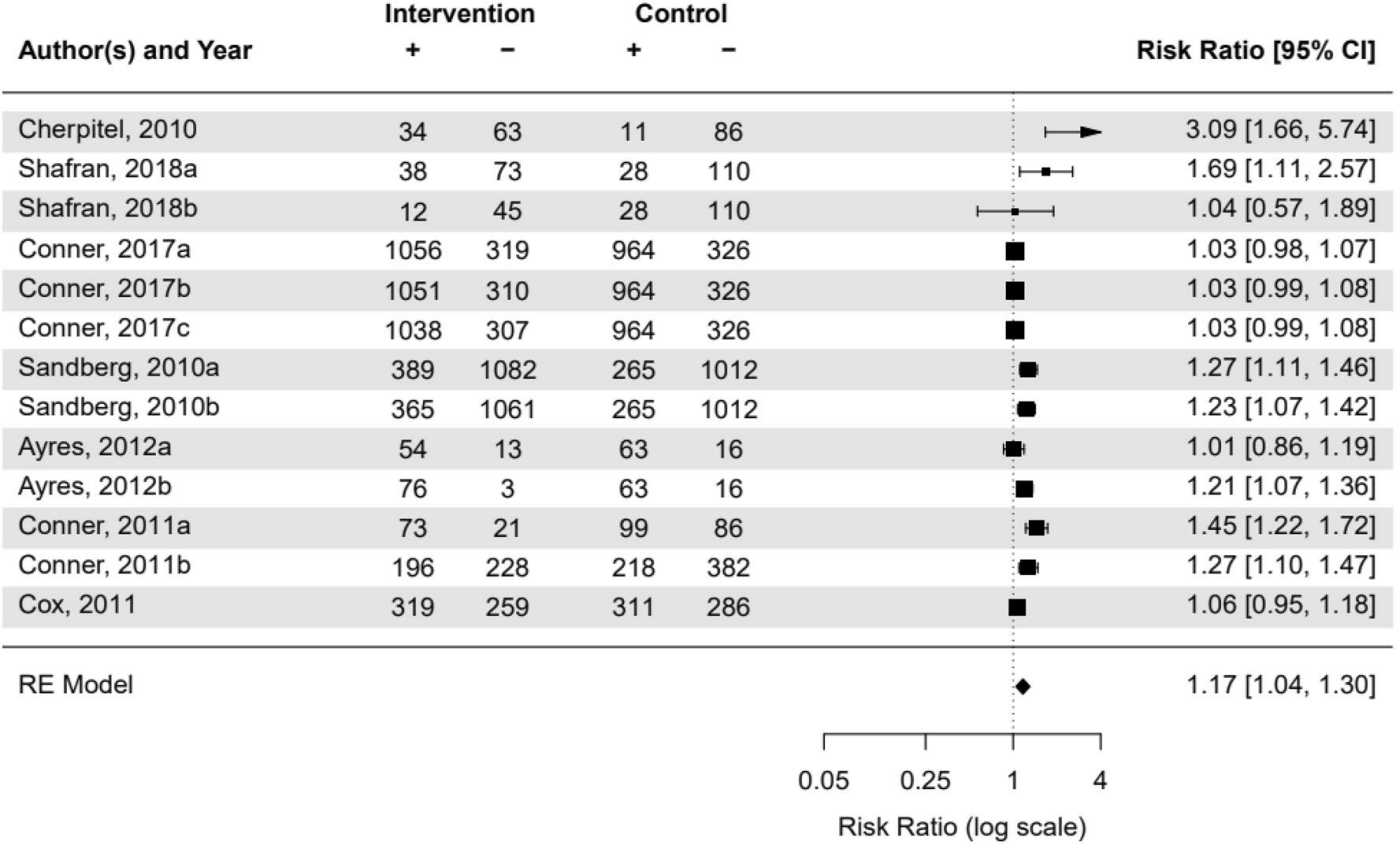
Meta-analysis of included studies: risk ratio of interventions. Notes: CI = confidence interval; RE = random effects

**Fig. 3:**
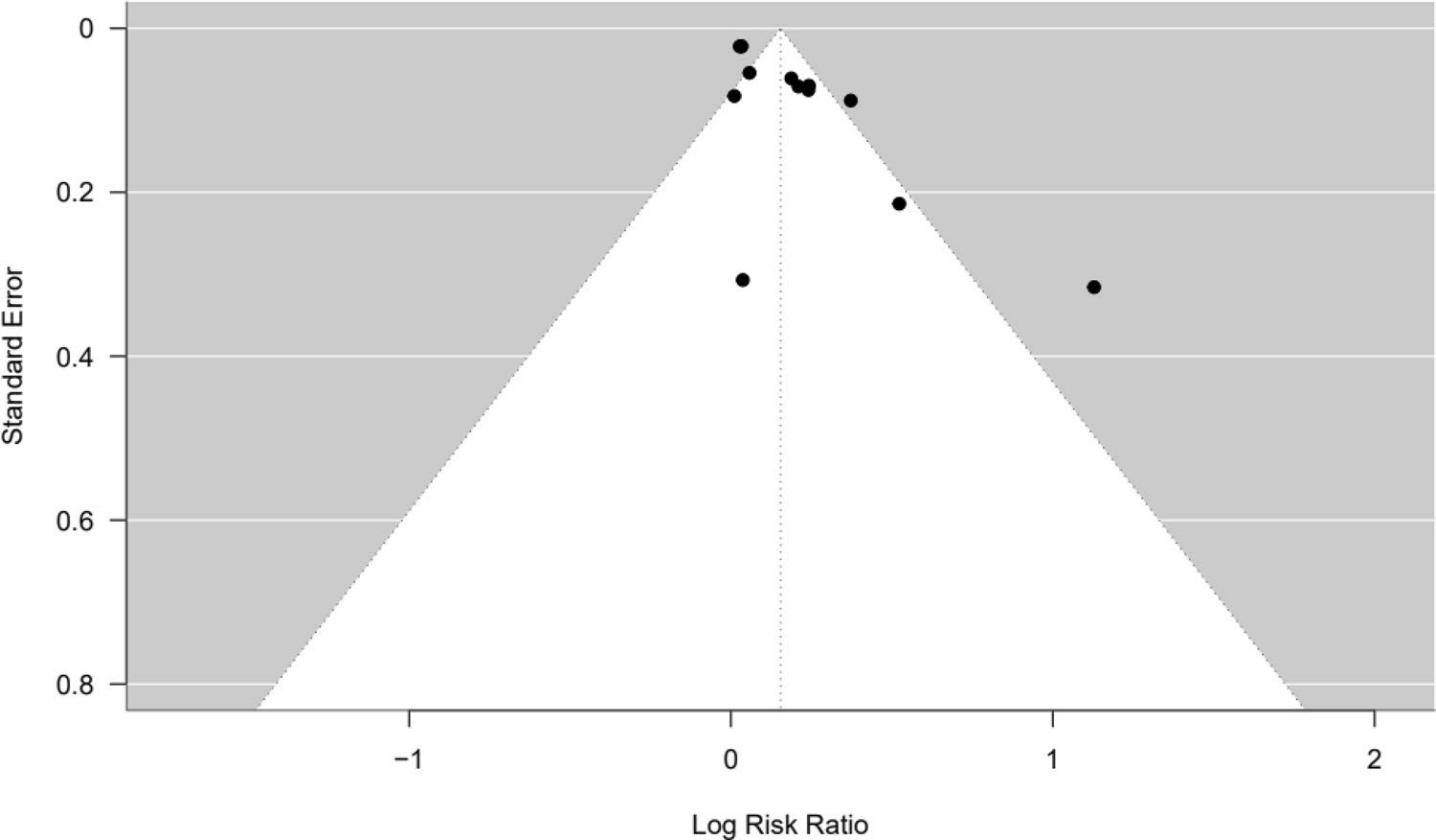
Heterogeneity of studies: statistical comparability of the interventions

Seven of the nine articles included a control condition, allowing for their inclusion in the meta-analysis of the risk ratio across interventions. From these 7 articles, 13 different interventions were assessed and included. There was a unidirectional, ubiquitously positive (health beneficial), and significant overall risk ratio of 1.17 [CI95% 1.04; 1.30] (see Fig. 2). The number of exposures and the emotional valence did not vary enough for inclusion in this analysis (87% of studies had only 1 exposure). Furthermore, the test for heterogeneity (τ^2^ = 0.037 (95%CI 1.04-1.30), p = 0.001) was significant, suggesting a lack of comparability across studies (Fig. 3).

## Discussion

A systematic review was conducted to examine the effects of measuring PROs on the responder’s item-related behaviours or perceptions In addition, the meta-analysis showed an overall risk ratio of 1.17 (see Fig. 2), indicating a health-beneficial effect of measurement on the outcome behaviour. A risk ratio of 1.17 means the risk of the outcome in the intervention group is 1.17 times (or 17% higher) than in the control group. For example, if 40% of a control group achieve the outcome naturally, the intervention group will have an expected probability of 40%×1.17=46.8%. While this is not a massive shift, and would not suggest that the mere-measurement effect could replace other forms of interventions or treatments, it does offer significant complimentary value. The results included nine articles and thirteen unique interventions. While there was a wide range of interventions and populations in the studies, an overall effect demonstrating an impact of taking measurements on the item-related perceptions or behaviors was found.

Notably, all studies reviewed delved into the mere-measurement effect of PROs without explicitly acknowledging it as such. Instead, many of the included studies referred to specific questionnaires they utilized, side-lining the term "PROs". This observation hints at the possibility that the mere-measurement phenomenon is more recognized in the field of cognitive psychology than within the domain of PRO experts which typically focus on psychometrics such as method effects.

In this study, we found that there is some evidence in the literature on PROs which hints towards the mere-measurement effect. For example, providing patients with access to elements such as survivorship care plans, incorporating PROs, may inadvertently negatively influence their overall quality of life [36]. This effect may stem from PROs heightening perceptions of illness threat, drawing patients' attention to potential side effects they might otherwise overlook [37]. This would not suggest that side effect queries should be avoided, but that they should be probed only when necessary. As illustrated by the study of Cartwright et al. (2012), this amplification may, in turn, influence the results of subsequent questionnaires or perceived impact of the disease [29]. Although not explicitly named as the mere-measurement effect, these initial studies provide valuable insight into this phenomenon. They underscore the imperative to investigate this effect collaboratively with PRO experts. If proven significant, it could emerge as a crucial factor to incorporate into future PRO studies as well as the development of PROs.

In the clinic, the mere-measurements positive effects could be harnessed to affect patient behaviours. PROs could be used as reminders in the clinic or through health applications to support treatment adherence or improve disease management. Responding to these outcomes acts an attentional director and accountability primer. PROs can also be constructed to elicit healthy intentions that the patients feel motivated to fulfil in the future, desiring to align their reported self with their true self. Employing the mere-measurement effect opens the door to PROs serving not only as outcomes but also as triggers and primers. A physical functioning scale could be used to prompt patients to move more. A disease management scale can alert patients to the need for action. In short, measurements can prime patients for positive behaviour change, nudge them to set healthy intentions, and remind them to take action.

Understanding the mechanisms underlying this phenomenon, psychologists, practitioners, and policymakers can devise novel strategies to influence behavior and decision-making, capitalizing on the subtle yet potent influence of measurement prompts. Consider a common outcome such as pain, it may be that measuring pain in fact increases pain perceptions over time as a result of regularly asking patients to focus their attention on their pain. If so, this would pave the way for the mere-measurement effect to be not only mitigated when deemed harmful, but also purposefully engineered to turn assessment into a positive intervention.

### Strengths and limitations

This is the first systematic review to the authors’ knowledge to assess the mere-measurement effect within the context of PROs. Despite the abstract nature of the mere-measurement effect, we have applied a robust methodology to navigate challenges and ensure the integrity of our findings. Four researchers participated in the title and abstract and full-text screening process to ensure a clear and comprehensive evaluation of appropriateness. This was needed as none of the reviewed articles explicitly employed PROs or PROMs as interventions, necessitating discussions on their inclusion in many instances. In addition, to establish clarity, operational definitions for key terms were meticulously formulated, and guidelines were predetermined and consistently adhered to. A decision was made to include only studies that explicitly targeted the mere-measurement effect or employed equivalent terminology, further enhancing the precision of our investigation.

Nonetheless a few limitations should be noted. This review did not identify any influence by emotional valence, but a larger sample with more variance could make a future assessment possible. Second, while the QA reviewers deemed the included articles to be generally high quality, a resulting majority of moderate assessments were due to a lack of methods transparency (incomplete reporting) rather than explicit weak design components. Further still, several of the included studies lack what can generally be categorized as a true control. Frequently, the control groups were exposed to potential contaminants, such as screening tools or other baseline assessments. Additionally, the meta-analysis revealed a significant lack of comparability, emphasizing the inconsistency and high diversity in interventions, outcomes, and populations assessed. Consequently, caution should be exercised in interpreting the results, given the potential for variability.

### Future directions

A randomized-controlled trial on the mere-measurement effect explicitly for PROs is the next step [38]. This will include a formal patient population and the use of validated and routinely used PROMs which assess disease-specific symptomology. The control will only include the primary outcome and no other secondary measures. Crucially, the mere-measurement outcome must be assessed observationally, i.e. without requiring subject response.

The studies presented also concentrated solely on utilizing the mere-measurement effect, but did not explore its potential harms. This can be accomplished without attempting to engineer a harmful questionnaire, but rather by including a standard scale which is negatively worded across every item.

The mere-measurement effect of PROs may present an opportunity to intentionally design assessments to serve as positive interventions. This systematic review identified studies demonstrating behavioural changes following the completion of questionnaires. Although most studies did not utilize validated PROMs, the findings underscore the need for additional exploration into their effect. Exploration into changes to the mere-measurement effect through item specificity should also be conducted. That is, does “I experience a lot of pleasure from jogging” have less of an effect than “I will experience a lot of pleasure from my jog this evening”. Item specificity has yet to be explored. The potential for assessments to induce positive changes in behaviour suggests a promising avenue for future research and intervention development.

The literature also suggests that the responder’s environment can have a significant impact on a resulting mere-measurement effect, although no study accounted for this. It may be that asking a patient about their treatment adherence in the care facility elicits a different effect than responding at home. This might be explained by the *context stability hypothesis.* The response environment - topic alignment should be incorporated in future experiments. As a final note, no mention of the mere-measurement effect in qualitative assessments were identified during initial literature investigation or throughout the study. This measurement format should also be evaluated in future research.

In sum, the mere-measurement effect acknowledges the multidimensional ways in which human cognition and behavior respond to the act of measurement. By understanding and identifying this phenomenon, psychologists, practitioners, and policymakers can devise novel strategies to influence health behavior and more importantly, minimize the negative effects of taking measurements. This sentiment is potentially even more important when considering vulnerable and stigmatized patient populations such as prisoners, victims of trauma, mother’s completing neonatal questionnaires, children or those struggling with addiction. Consider, for example, the potential for a harmful mere-measurement effect when asking an individual trying to quit smoking to respond to the item *‘I want to have a cigarette’*. Our goal as medical professionals must be to search for the best balance of data quality and patient impact. As our awareness of this phenomenon grows, so does its potential.

## Conclusion

This review provides the first evidence of the significant potential for the mere-measurement effect to influence patients through PROs. However, the small number and heterogeneity of the included studies highlight the need for more focused and systematic research on the mere-measurement effect on PROS, with repeated exposures and varying emotional valence, ideally through a randomized controlled trial. Both clinicians and researchers must be aware of the implicit influence that measuring PROs can have on patients’ actions, as this effect may have important implications on the future use of PROs.

## Supplementary Information

Below is the link to the electronic supplementary material.

Supplementary file1 (DOCX 36 kb)

## Data Availability

Data and data collection forms from the systematic review can be acquired through reasonable request of the corresponding author. The protocol was published in in medRxiv (medRxiv 2022.03.29.22273094; 10.1101/2022.03.29.22273094).
